# Teleoncology: The Youngest Pillar of Oncology

**DOI:** 10.1200/GO.20.00295

**Published:** 2020-09-30

**Authors:** Puneet Pareek, Jeewan Ram Vishnoi, Sri Harsha Kombathula, Rakesh Kumar Vyas, Sanjeev Misra

**Affiliations:** ^1^ Department of Radiation Oncology, All India Institute of Medical Sciences, Jodhpur, India; ^2^ Department of Surgical Oncology, All India Institute of Medical Sciences, Jodhpur, India

## Abstract

The core pillars of multimodal care of patients with cancer are surgical, radiation, and medical oncology. The global pandemic of coronavirus disease 2019 (COVID-19) has suddenly resurrected a new pillar in oncology care: teleoncology. With oncologists reaching out to patients through telemedicine, it is possible to evaluate and fulfill patients’ needs; triage patients for elective procedures; screen them for influenza-like illness; provide them with guidance for hospital visits, if needed; and bridge oral medications and treatments when a hospital visit is not desirable because of any high risk-benefit ratio. Teleoncology can bring great reassurance to patients at times when reaching an oncology center is challenging, and more so in resource-constrained countries. Evidence-based treatment protocols, dispensable by teleoncology, already exist for many sites of cancer and they can provide a bridge to treatment when patients are unable to reach cancer centers for their standard treatment. The young pillar of teleoncology is going to remain much longer than COVID-19.

## INTRODUCTION

Cancer is one of the leading cause of disability and death worldwide. An estimated 18.1 million people are diagnosed with cancer each year, of which 9.6 million die of their disease.^1^ India is inhabited by >17% of the world’s population. An estimated 1.1 million people are diagnosed each year with cancer in India. Unlike the populations in Western countries, patients are diagnosed at later stages and a decade younger.^2^ The 10 topmost cancers affecting the disability-adjusted life years in India are those of the stomach, breast, lung, lip and oral cavity, pharynx other than nasopharynx, colon and rectum, leukemia, cervix, esophagus and brain, and nervous system.^3^

## MULTIMODAL CARE IN ONCOLOGY

As medicine evolved, solid tumors that were amenable to resection were attempted to be managed by increasingly aggressive surgeries. In the 19th century, William Halsted, from John Hopkins University in the United States, established such procedures as radical mastectomy, in which breast, muscles, and even ribs were removed in pursuit of control of breast cancer.^4^ The high rates of relapse, despite such morbid procedures and challenges to manage the disseminated disease, prompted clinicians involved in the care of patients with cancer to incorporate other evolv-ing modalities, such as radiation therapy, hormonal therapy, and chemotherapy. Bertrand Fischer proposed the systemic paradigm of cancer and, in a series of meticulous landmark studies, increased the survival of patients with breast cancer using multimodal care and less morbid surgeries.^5^Gradually, the three pillars of oncology were identified as surgical oncology, dealing with surgeries; and other two nonsurgical pillars of radiation oncology and medical oncology, making use of local radiation therapy and systemic therapies, respectively. Almost all patients with cancer need multimodal care with complex sequencing and synergistic combinations as laid down by biological rationale or evidence from clinical trials. Systems evolved in various hospitals in which either the patients are seen jointly by clinicians from all contributing specialties or, if seen already, are discussed and a multimodal care plan is formulated at the tumor boards. Tumor boards are multidisciplinary team meetings in which all contributing clinicians make decisions and chart the care plan of the patient. As demonstrated by numerous systematic reviews, the multidisciplinary team approach is the best strategy for the care of patients with cancer.^6-8^

## CORONAVIRUS DISEASE 2019 AND PROBLEMS IN CANCER CARE

Novel coronavirus disease 2019 (COVID-19) caused by SARS-CoV-2 appears to have originated in December 2019 from Wuhan, Hubei, China, and in January 2020, WHO declared the same a global pandemic. The pandemic brought a Pandora’s box of problems. There was a problem of travel and resources because of lockdowns imposed to control the spread of disease. The rapid wave of the infection consumes hospital resources, including both the workforce, which is either diverted to take care of patients with COVID-19 or gets quarantined thereafter, and equipment and beds, which cannot be shared with noninfected patients. Patients with cancer are being affected by all the problems posed by COVID-19 and, in addition, are a high-risk population for the disease because of their immunocompromised status. Although the world saw the plight of China in advance, populations of most countries have gradually become affected at different rates and at different times.^9-12^

### COVID-19 and Cancer Care Guidelines

As the wave of COVID-19 infection swept across countries, there were many dilemmas. Many elective procedures by various medical specialties were halted to divert resources for COVID-19 care; emergency care, however, continued. Oncology patients of different age groups and different disease stages occupy a whole spectrum of priority where their segregation into a simple binary group of elective versus the emergency need of treatment gets blurred. Thus, the treatment at most cancer centers has continued, even though other specialties paused in response to COVID-19 at most centers. Various oncology societies and eminent, comprehensive cancer centers rapidly issued guidelines and are revising them as and when needed because of evolving scenarios due to COVID-19.^13-20^ Most of the guidelines insist on assigning different priorities to patients, with the highest priority assigned to rapidly proliferating tumors in patients treated with curative intent, and patients expected to have maximum disease-free status. The lowest priority is for tumors that either proliferate slowly, and delay in treatment is not expected to cause any compromise in outcomes, or advanced stage tumors in frail and elderly patients in whom any extension of life is highly improbable.

### Oncosurgery and COVID-19

Postoperative outcomes in patients infected with SARS-CoV-2 are substantially worse than prepandemic baseline rates of pulmonary complications and mortality.^21^ Appreciating the highly variable COVID-19 conditions across different regions, the American College of Surgeons has proposed three acute phases and two recovery phases, as follows^22^:

- Acute phase I: semi-urgent setting (preparation phase): Surgery restricted to patients likely to have survivorship compromised if surgery is not performed within the next 3 months.
- Acute phase II: urgent setting: Surgery restricted to patients likely to have survivorship compromised if surgery is not performed within the next few days.
- Acute phase III: exhausted setting: Surgery restricted to patients likely to have survivorship compromised if surgery not performed within the next few hours.
- Early recovery phase: Past the peak of COVID-19, with fewer new cases recorded each day.
- Late-phase recovery: Well past the peak of new COVID-19 cases by at least 14 days.

Besides patient care, all guidelines also emphasized managing of staffing, the preparedness of the hospitals, infection control, and scheduling.

## TELEONCOLOGY, THE YOUNGEST PILLAR IN SUPPORT DURING CRISIS

The oncology community is armed with various guidelines that prioritize patient care. However, there are two more problem groups remaining: (1) the problems due to lockdown, and (2) issues related to social distancing. The lockdown affects the travel of patients, and social distancing affects physical multidisciplinary-team meetings. Most patients are unable to travel to their primary oncology centers. In the low- and middle-income countries, often there are no oncology services near patients’ homes.^23-25^ A diagnosis of cancer, treatment interruption, and no contact with oncologists constitute a cocktail for suffering. Telemedicine using various modes of communication between the patient and health care providers, as well as among different health care specialists for joint care, has been promptly resurrected as one of the responses to the COVID-19 crisis.^26-29^ Telemedicine has appeared as a strong and long-lasting pillar of oncology.^30-34^

Telemedicine involves the use of any audiovisual information technology for interaction or monitoring of individuals or communities for health purposes. WHO has adopted the definition of telemedicine as “The delivery of health care services, where distance is a critical factor, by all health care professionals using information and communication technologies for the exchange of valid information for diagnosis, treatment and prevention of disease and injuries, research and evaluation, and for the continuing education of health care providers, all in the interests of advancing the health of individuals and their communities”^35^. Laws in most countries had some restrictive attitude toward telemedicine, with an apprehension to protect people from medical errors in the absence of direct interaction. However, with the embracing of telemedicine borne out of urgent need due to COVID-19, there have been rapid changes in such laws, with the adoption of various frameworks of telemedicine.^28,33,36^ The information technology paraphernalia is flooded by multiple tools like android and iOS applications for chatting and video calls, chatbots with artificial intelligence, and various software (eg, Apple’s Facetime; Apple, Cupertino, CA), Skype (Microsoft, Richmond, WA), and Cisco Webex (CISCO, San Jose, CA), among others).

The telemedicine technological base cannot be generalized and is best chosen by the bottom-up approach: when providing services to the patient, always choose what the patient can access. Hospitals or health care professionals using their superior technological tools with patients is often a reason for poor compliance of patients to telemedicine.^37,38^ Simple phone calls, text messages, and popular android applications readily available to patients should be used for audio-, text-, or video-based services in telemedicine. Any multidisciplinary team discussions and academics can use higher technologies with video conferencing, and often presentation and screen sharing, available to health care workers and medical trainees. A uniform and ethical structure of telemedicine consultation and prescription usually takes care of the prevention of errors. Telemedicine guidelines issued by various medical council and regulatory authorities have drafted mandatory structure of such prescriptions like a clearly written name, age, drugs prescribed, date of prescription, and doctor’s name with certification and registration details, and so forth.^36^

Teleoncology during COVID-19 is serving multiple objectives (Table 1). Patients planned for hospital admission and procedures can be screened by teleoncology for any signs and symptoms of influenza. They can be guided toward the hospital protocol for treating patients suspected of having COVID-19 instead of a surprise finding in busy day care scheduled for the next cycle of chemotherapy or radiation therapy. Those who are not suspected of having influenza-like illness can often get baseline investigations done close to their home and then get instructions on how to visit in the hospital and schedule their appointments. Patients receiving routine follow-up who usually do not need radiology investigations, unless symptomatic, can avoid hospital visits, and teleoncology can suffice.

**TABLE 1:**
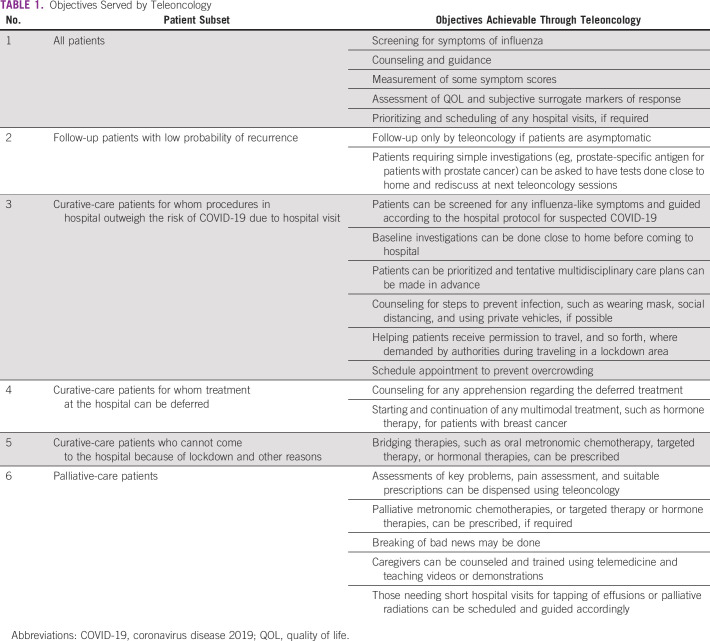
Objectives Served by Teleoncology

Prioritizing can also be done through a teleoncology consultation. Patients who require active oncology treatment but are either unable to visit the hospital or stay for the required period close to the hospital can be often prescribed bridging multimodal treatments like metronomic oral chemotherapies and hormone therapies before they can finally visit the cancer center for the standard-of-care treatment of their particular cancer. Where treatments can be safely deferred, like postoperative adjuvant radiation in hormone-positive early breast cancer, the same can be communicated and counseled using teleoncology. Patients treated with palliative intent who travel unnecessarily during the time of COVD-19 have a high risk-benefit ratio. It perfectly justifies treating these patients and issuing prescriptions by teleoncology except when the patient needs to visit the hospital for short procedures like tapping of effusions, insertion of a nasogastric tube, or palliative radiation therapy, which can be delivered as a single or hypofractionation to reduce their visits to the hospital. Palliative metronomic chemotherapies can also be prescribed through teleoncology. Extremely emotionally challenging tasks such as communicating bad news and even providing bereavement care may also be done through teleoncology.^39,40^

## TELEONCOLOGY ADVANTAGES AND LIMITATIONS

In addition to being a strategic tool for infection control for patients, caregivers, and health care professionals, teleoncology saves the cost of traveling and lodging near hospitals.^30^ On a teleoncology platform, caregivers and relatives who are staying at different places and even specialists who are otherwise not available in the parent cancer center may be invited for joint care-plan formulations and counseling.^41^ Systematic reviews, although unable to identify studies with large numbers of patients, did show that the cost of consultation was reduced with no perception of impaired ability to decide on care plans.^42^ However, there may be patients as well as health care professionals who may be unable to perceive empathetic communications using only electronic tools, as compared with personal contact.^43^ Patients with cognitive disabilities may not be best suited for any telemedicine assessment but, at the same time, may benefit by avoiding unnecessary travel.^44^Moreover, the most important objective of teleoncology is triage of suitable patients. In the absence of proper triage, patients in real need of a personal visit to the cancer center may swap spaces with patients not requiring the same and this may waste time and resources of all stakeholders. In low- to middle-income countries, the pharmacy chains are complicated and usually not electronically linked to hospitals. There is lack of trained pharmacists and, in remote areas, often untrained personnel may be at the retail counters of pharmacies.^45^ An uneducated patient with an untrained pharmacist may not be the best combination for dispensing telemedicine prescriptions; thus, misadministration of drugs and dosages is a real challenge.^46,47^

## TELEONCOLOGY IN COMMON CANCERS

Patients with cancers of the breast, lung, and head and neck constitute a large portion of patients who require recurring care and visits to the oncology centers. Besides triage, counseling and follow-up, as listed in Table 1, there are an abundance of evidence-based oral medication protocols that may be used for radical and palliative care and as bridging prescriptions through teleoncology. In patients with hormone receptor–positive breast cancer, oral hormonal therapies can be a safe substitute for neoadjuvant and adjuvant chemotherapy.^48,49^ Similarly, for hormone receptor–negative breast cancers, oral chemotherapies may be used.

In oral cancers, all efforts are to be made to provide the definitive treatment modality without unnecessary delay. The guidelines on the management of head and neck cancers during COVID-19 by American and European societies like the American Society for Radiation Oncology and the European Society of Therapeutic Radiology and Oncology suggest that no delay is acceptable in initiating radical treatment.^50^ For patients who are unable to reach the hospital to receive intravenous chemotherapy, which might be the case in low- to middle-income countries where patients are unable to travel during COVID-19, oral methotrexate is an attractive option. In a matched-pair analysis done at Tata Memorial Hospital, metronomic methotrexate as a neoadjuvant therapy in head and neck cancers resulted in an improvement in disease-free survival.^51^ Similarly, patients with metastatic lung cancer with epidermal growth factor–receptor mutations and patients with chronic myeloid leukemia with *BCR-ABL* mutations can receive oral targeted agents and be easily monitored by teleoncology. Thus, evidence-based interventions are available for patients who are either unable or are not required to visit the cancer cen-ter in person.In conclusion, the treatment of patients with cancer has evolved gradually to be multimodal care. The COVID-19 pandemic has suddenly resurrected a new pillar of oncology care—teleoncology—proving a novel way to ensure patients’ and health care providers’ needs are met. With the oncologists reaching patients at home via telemedicine tools, it is possible to evaluate patients’ needs; triage patients for elective procedures; screen them for influenza; provide them guidance for hospital visits, if needed; and bridge oral medications and treatments when a hospital visit is not desirable because of any high risk-benefit ratio. The rules of various regulatory bodies have become supportive regarding telemedicine, and this often can bring great reassurance to patients at times when reaching the oncology center is challenging, and even more so in resource-constrained countries.

Interestingly, evidence-based treatment protocols already exist for many such bridge treatments, which can be prescribed through teleoncology. The judicious use of such teleoncology protocols becomes a part of personalized oncology care. Oncologists are integrating the teleoncology pillar to strengthen support to their patients—a pillar that is going to be around longer than COVID-19.
